# Exploring the Impact of Ampelopsis Grossedentata Flavonoids on Growth Performance, Ruminal Microbiota, and Plasma Physiology and Biochemistry of Kids

**DOI:** 10.3390/ani13152454

**Published:** 2023-07-29

**Authors:** Junhong Zhu, Xingneng Liu, Ying Lu, Dan Yue, Xiaoming He, Weidong Deng, Sumei Zhao, Dongmei Xi

**Affiliations:** 1 Yunnan Provincial Key Laboratory of Animal Nutrition and Feed, Faculty of Animal Science and Technology, Yunnan Agricultural University, Kunming 650201, China; junhong-zhu@foxmail.com (J.Z.); 18787055931@163.com (X.L.); yinglu_1998@163.com (Y.L.); danyue0528@foxmail.com (D.Y.); xiaominghe@foxmail.com (X.H.); dengwd@ynau.edu.cn (W.D.); 2 Institute of Animal Husbandry, Yunnan Vocational College of Agriculture, Kunming 650201, China

**Keywords:** growth performance, Ampelopsis grossedentata flavonoids, ruminal microbiota, kids

## Abstract

**Simple Summary:**

In the face of the challenge posed by antibiotic prohibition, the identification of sustainable, eco-friendly alternatives to antibiotics is important. Ampelopsis grossedentata flavonoids, a plant-based extract, have high antioxidant and anti-inflammatory activities provide a non-toxic and innocuous option for the extended application of additives. This study examined the viability of Ampelopsis grossedentata flavonoids as a potential substitute for antibiotics. The results showed that supplementation of Ampelopsis grossedentata flavonoids in the diet exhibited the capacity to enhance rumen microbial diversity and immune competence, thereby fostering improved growth performance among weaned kids. Consequently, this study provides an important reference for the future development of Ampelopsis grossedentata flavonoids as a new environmentally friendly additive.

**Abstract:**

This study was conducted to evaluate the influences of supplementing Ampelopsis grossedentata flavonoids (AGF) on the rumen bacterial microbiome, plasma physiology and biochemistry, and growth performance of goats. Twenty-four Nubian kids were randomly allocated to three dietary treatments: the control (CON, basal diet), the 1.0 g/kg AGF treatment (AGF), and the 12.5 mg/kg monensin treatment (MN). This trial consisted of 10 days for adaptation and 90 days for data and sample collection. The results reveal that *Bacteroidetes*, *Firmicutes*, and *Proteobacteria* are the dominant phyla in kids’ rumen. Compared with the CON group, the alpha diversity in the MN and AGF groups significantly increased (*p* < 0.01). Beta-diversity shows that rumen microbial composition is more similar in the MN and AGF groups. LEfSe analysis shows that *Prevotella_1* in the AGF group were significantly higher than those in the MN and CON group. The high-density lipoprotein cholesterol and glucose levels in the AGF group were significantly higher than those in the CON group (*p* < 0.05), whereas the low-density lipoprotein cholesterol, glutamic-pyruvic transaminase, and alkaline phosphatase levels exhibited the opposite trend. The average daily gains in the AGF and MN groups significantly increased, while the feed-to-gain ratios were significantly decreased (*p* < 0.05). The results suggest that adding AGF to the diet improves microbial composition and has important implications for studying juvenile livestock growth and improving economic benefits.

## 1. Introduction

Ampelopsis grossedentata has a history of hundreds of years in China as a health-promoting tea and herbal medicine [1]. Ampelopsis grossedentata flavonoids (AGF) are natural plant extracts derived from Ampelopsis grossedentata, with the primary component being dihydromyricetin [2]. These flavonoids exhibit high antioxidant and anti-inflammatory activities. Studies have found that AGF can eliminate nitrites within the animal body, and long-term consumption poses no toxic effects [3,4,5,6]. Animal studies indicate that Ampelopsis grossedentata can effectively decrease serum total cholesterol levels, minimize lipid accumulation, and prevent and alleviate hyperlipidemia and hyperglycemia-related metabolic disorders [7,8]. Recent reports indicate that AGF holds great promise as a therapeutic option for obesity and its associated metabolic disorders, as demonstrated by its remarkable ability to curtail body weight gain, reduce liver weight, and mitigate abdominal fat accumulation in mice afflicted with non-alcoholic fatty liver disease [9]. Taking this knowledge further, Li Fan delved into the intricate interplay between dihydromyricetin and the gut microbiota of rats, using 16S rRNA pyrosequencing to gain deeper insight into the long-term effects of this flavonoid. His results revealed a significant modulation of gut microbiota abundance, diversity, and composition, illuminating a promising avenue for the development of novel therapeutics [10].

Previous studies have shown the hepatoprotective effects of Ampelopsis grossedentata extract (AGE) against acute liver injury in mice induced by the notoriously toxic agent CCl_4_ [11]. The salutary actions of AGE are attributed to its ability to counteract liver inflammation, avert intestinal epithelial barrier damage, and restore gut microbiota dysbiosis to homeostatic levels. Remarkably, the application of AGF as potential feed additives in ruminant animals remains underexplored. Monensin (MON) is an ion carrier antibiotic with the potential to regulate rumen fermentation and improve animal performance [12,13]. As an antimicrobial supplement, monensin has demonstrated its ability to enhance daily weight gain, feed conversion efficiency, proportion of lean meat, and area of the eye muscle in ruminant animals [14,15,16]. It occupies an important position in the animal husbandry industry and brings great convenience and economic benefits to people. However, the use of antibiotics in food animal production has been banned due to the risk of accumulation of residues in animal products and the emergence of antibiotics-resistant bacterial strains [17,18,19]. Several challenges are associated with the withdrawal of antibiotics from feeds [20,21]. Therefore, finding sustainable and environmentally friendly alternatives to antibiotics is particularly important.

Here, we aimed to comprehensively investigate the influences of dietary AGF addition on the growth performance, ruminal microflora, and plasma physiology and biochemistry of Nubian kids, so as to evaluate the effectiveness of AGF as a replacement for monensin and provide a theoretical foundation for their future implementation in the ruminant industry.

## 2. Materials and Methods

### 2.1. Animals, Diets, and Experiment Design

In a single-factor experimental design conducted in Yunnan Province, China, twenty-four healthy, simultaneously weaned Nubian kids (mean initial weight: 14.57 ± 0.81 kg of body weight, two months old) were chosen for the investigation. These subjects were then randomly and evenly allocated into three distinct groups, each consisting of eight individuals (half male and half female). The dietary regimens employed were devised by the NRC (2007) and NY/T816-2004 guidelines for basal diet (Table 1). The content of aflatoxin in corn was checked, and the content was 0–0.8 μg/kg, which was adequate [22].

The control group (CON) received a basal diet, while the test groups were administered either a basal diet supplemented with 12.5 mg/kg monensin (MN group) or a basal diet containing 1.0 g/kg Ampelopsis grossedentata flavonoids (AGF group). All the kids were fed ad libitum twice per day at 06:00 h and 16:00 h with free access to fresh water. There was no difference between the three groups. The entire experiment was 100 days, comprising 10 days of adaptation and 90 days of the feeding period. All the animal manipulations were approved by the Ethics Committee of Experimental Animals of Yunnan Agricultural University.

### 2.2. Growth Performance

At predetermined time points (07:00) on days 11, 41, 71, and 101, kids underwent body weight assessments following a fasting period. The feed intake for each group was scrupulously recorded, facilitating the accurate determination of critical parameters such as initial body weight (IBW), final body weight (FBW), average daily feed intake (ADFI), average daily gain (ADG), and feed-to-gain ratio (F/G).

### 2.3. 16S rRNA Gene Sequencing

On day 101 of the investigation, preprandial rumen fluid was procured utilizing a vacuum pump pressure apparatus, and six representative kids from each of the three cohorts were chosen based on their proximity to the average group weight. High-throughput sequencing, amplifying distinctive microbial sequences in the 16S region, were conducted for microbial detection [23]. Microbial genomic DNA extraction from individual samples was carried out via the HiPure Stool Soil DNA Kit (Meiji, D3141, Guangzhou, China), adhering to the manufacturer’s guidelines. Quantification of DNA was achieved using a NanoDrop 2000 spectrophotometer (Thermo Fisher Scientific, Madison, WI, USA) to ensure the isolation of sufficient high-quality genomic DNA. PCR amplification of the V3-V4 region of the 16S rRNA genes was executed utilizing the forward primer (5′-CCTACGGGNGGCWGCAG-3′) and the reverse primer (5′-GGACTACHVGGGTATCTAAT-3′). The resulting PCR amplicons underwent paired-end sequencing on an Illumina MiSeq platform (Gene Denovo Biotechnology Co., Ltd., Guangzhou, China) according to the established protocol (PE250).

The raw FASTQ data files underwent processing for duplicate elimination, quality filtration, and analysis employing QIIME 1.8.0 (Quantitative Insights Into Microbial Ecology) [24,25,26,27], in accordance with the standard delineated by Mao et al. [28]. High-quality sequencing fragments were conserved for subsequent data examination. Operational taxonomic units (OTUs) were reclustered utilizing UPARSE software (version 7.1) with a 97% sequence similarity threshold [29], and chimeric sequences were discerned and eliminated via the Uchime algorithm [30,31].

Alpha diversity analysis was executed utilizing QIIME software (version 1.9.1), while beta diversity was represented by the PCoA index [32]. Species exhibiting a relative abundance of at least 0.1% in one sample were depicted using the R language circlize package (version 1.6.16). Nonmetric multidimensional scaling (NMDS) was generated through the R project vegan package (version 2.5.3) and plotted using the R project ggplot2 package (version 2.2.1) [33,34]. Intergroup Venn analysis was conducted with the R project VennDiagram package to discern unique and common species [35]. LEfSe software (version 1.0) facilitated the screening of biomarker features in each group [36]. KEGG pathway analysis of the OTUs was inferred via Tax4Fun (version 1.0) [37].

### 2.4. Plasma Physiology and Biochemistry

At predetermined time points (07:00) on day 101, blood specimens were collected from the jugular veins of eight individuals per group into vacutainer tubes. Subsequently, samples underwent immediate centrifugation at 3000 rpm and 4 °C, with plasma being extracted and preserved at −20 °C until further analysis. Plasma physiology and biochemistry, encompassing total protein (TP), albumin (ALB), globulin (GLOB), total cholesterol (TC), triglyceride (TG), high-density lipoprotein cholesterol (HDL-CH), low-density lipoprotein cholesterol (LDL-CH), glucose (GLU), alkaline phosphatase (ALP), lactate dehydrogenase (LDH), glutamic-oxaloacetic transaminase (GOT), and glutamic-pyruvic transaminase (GPT), were quantified employing an automated biochemistry analyzer (Roche, Basel, Switzerland).

### 2.5. Data Analysis

The acquired data were preliminarily processed within an Excel spreadsheet and subsequently subjected to one-way ANOVA utilizing SPSS 25.0 statistical software, with results presented in tables as means and pooled standard errors of the means (SEM) [38]. Multiple comparisons of means employed LSD and Duncan methodologies, with disparities established based on *p* values. Extremely significant differences were denoted by *p* < 0.01, significant differences by *p* < 0.05, and non-significant differences by *p* > 0.05.

## 3. Results

### 3.1. Growth Performance

The ADGs of the AGF and MN groups were higher than the CON group (*p* < 0.05), which improved by 28.05% and 22.57%, respectively, compared to the CON group. The F/Gs of the AGF and MN groups were lower than the CON group (*p* < 0.05) and reduced by 38.71% and 34.28%, respectively, compared to the CON group (Table 2).

### 3.2. Composition and Diversity of the Rumen Microbiota

#### 3.2.1. Rumen pH

Rumen pH values were ascertained for the CON group, MN group, and AGF group, with values of 6.77 ± 0.07, 6.77 ± 0.12, and 6.75 ± 0.23, respectively. The distinct additives exhibited no significant impact on rumen pH in kids (*p* > 0.05).

#### 3.2.2. Composition of the Rumen Microbiota

After the removal of tags indicating low quality or no biological significance, a total of 2,149,941 effective tags were acquired from the 18 samples analyzed. Each sample was covered by an average of 119,441 effective tags. The effective ratio of all samples averaged 93.25% (ranging from 90.82% to 95.00%) (Table S1). The sequencing results represent the true situation of microbiota because the Good’s coverage values of all samples averaged 99.82% (ranging from 99.76% to 99.89%) (Table S2).

**Figure 1:**
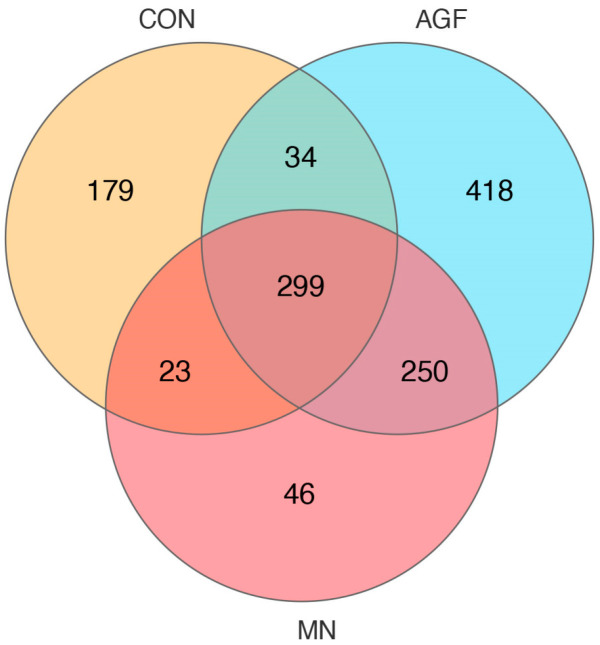
Venn diagram at the OTU level of rumen microbiota in three groups.

**Figure 2:**
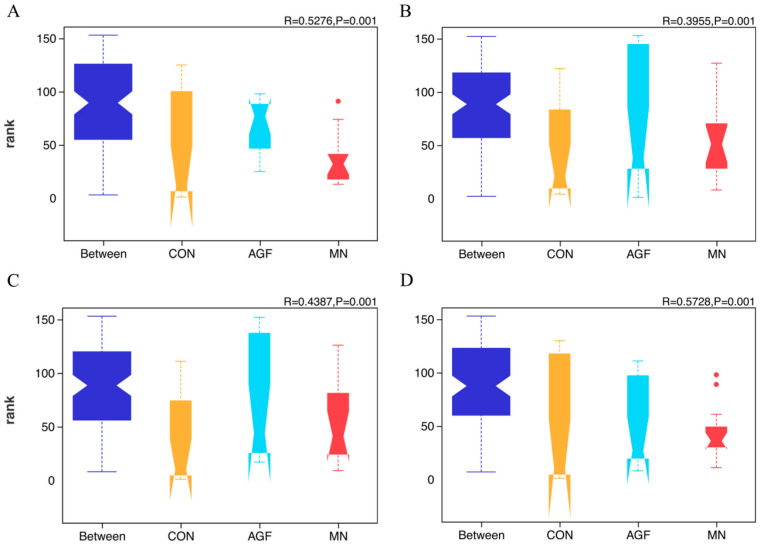
ANOSIM analysis at the OTU (**A**), phylum (**B**), family (**C**), and genus (**D**) levels of rumen microbiota composition in three groups.

**Figure 3:**
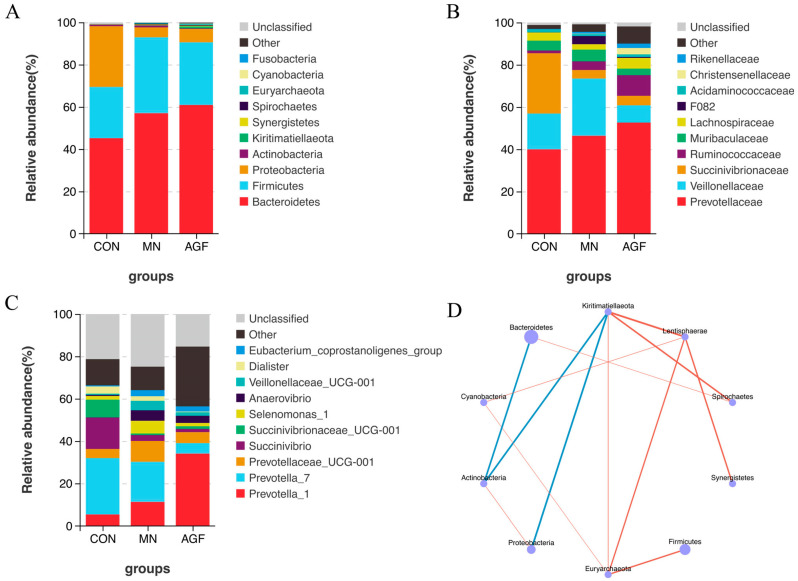
Species composition analysis. (**A**) Top 10 dominant species at phylum level. (**B**) Top 10 dominant species at family level. (**C**) Top 10 dominant species at genus level. (**D**) Phylum level species correlation network diagram (*p* < 0.05). The blue line in (**D**) represents a negative correlation, and the red line represents a positive correlation.

A total of 299 operational taxonomic units (OTUs) were shared by three groups, whereas 418 OTUs, 46 OTUs, and 179 OTUs were unique to the AGF group, MN group, and CON group, respectively (Figure 1). The compositions of the rumen microbiota in the AGF, MN, and CON groups were significantly different at the OTU, phylum, family, and genus levels (*p* < 0.01; Figure 2). At the phylum level, the main rumen microbiota included *Bacteroridetes*, *Firmicutes*, and *Proteobacteria*, which together account for approximately 95% of the total microbiota population, with relative abundances ranging from 45% to 61%, 24% to 36%, and 4% to 29%, respectively (Figure 3A; Table S3). The relative abundance of *Proteobacteria* was significantly lower in the AGF and MN groups compared to the CON group (*p* < 0.01). No significant differences were observed among the other phyla in the different groups (*p* > 0.05) (Table S3).

At the family level, *Prevotellaceae* was the dominant microbiota family (Figure 3B). The relative abundance of *Succinivibrionaceae* was significantly higher in the AGF and MN groups compared to the CON group (*p* < 0.01). Meanwhile, the relative abundance of Ruminococcaceae was significantly lower in the AGF group compared to the CON group (*p* < 0.05) (Table S4). At the genus level, compared to the CON group, the relative abundance of the *Prevotella_1* of the AGF and MN groups was significantly higher (*p* < 0.05), with *Prevotella_7* being on the contrary. The relative abundance of *Succinivibrio* was significantly lower in the AGF and MN groups compared to the CON group (*p* < 0.01). The relative abundance of *Dialister* was significantly lower in the AGF group compared to the CON group (*p* < 0.05) (Figure 3C; Table S5). All correlations among the top 10 dominant species are shown. There was a significant negative correlation between *Actinobacteria*, *Bacteroides*, and *Kiritimatiellaeota* and a significant positive correlation between *Lentisphaerae*, *Euryarchaeota*, and *Cyanobacteria* (Figure 3D).

#### 3.2.3. Diversity of the Rumen Microbiota

**Figure 4:**
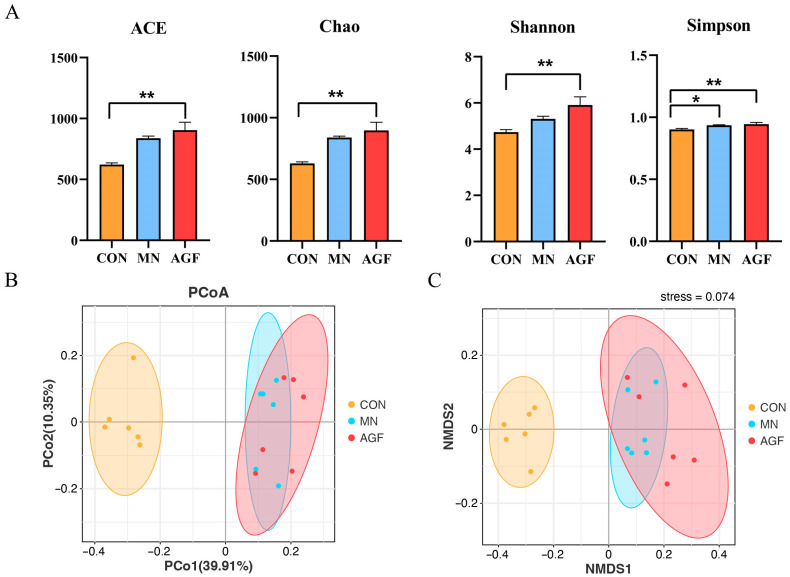
Diversity of rumen microbiota community as affected by additives. (**A**) ACE, Chao, Shannon, and Simpson indexes; (**B**) PCoA analysis; (**C**) NMDS ordination. * Indicates significant difference (*p* < 0.05), and ** indicates extremely significant difference (*p* < 0.01).

As can be seen, the above four alpha-diversity indexes in the AGF group were higher than those of the CON group (*p* < 0.01) and MN group (*p* < 0.05) (Figure 4A). These results indicate that the AGF group had the highest species richness and evenness in the rumen fluid of kids and therefore the highest species diversity as well. The beta diversity, evaluated by principal coordinate analysis (PCoA) utilizing taxonomic data at the OTU level, disclosed conspicuous distinctions among the rumen microbiota communities in the three groups. In the PCoA plot, coordinates 1 and 2 account for 39.91% and 10.35% of the total variance, respectively (Figure 4B). The stress value is calculated as 0.074, and intra-group sample points exhibit consistency (Figure 4C). Notably, the rumen microbial composition was more similar in the MN and AGF groups.

#### 3.2.4. Analysis of Rumen Differential Microbiota

**Figure 5:**
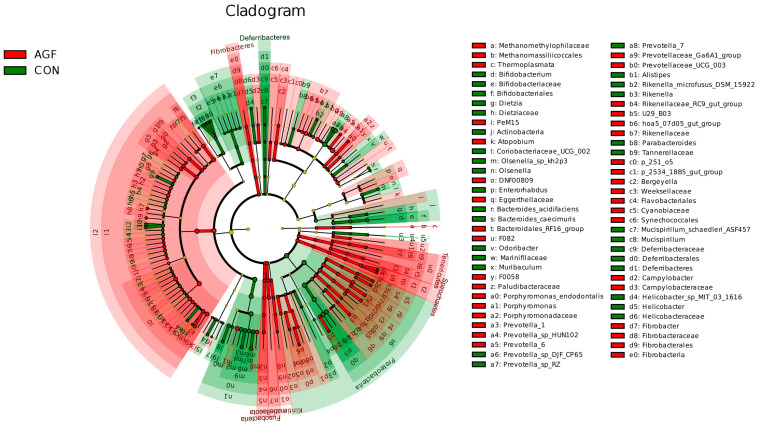
LEfSe analysis of indicator species.

Different additives biomarkers with statistical differences between groups were searched for by LEfSe analysis. A total of 239 biomarkers with different taxonomic levels were found in the AGF and CON groups, *Prevotella_1* and *Prevotella_7* were the important taxa contributing to the differences in the rumen microbiota between the AGF and CON groups (Figure 5, Table S6). A total of 213 biomarkers were found in the MN and CON groups, and *Succinivibrionaceae_UCG_001* and *Prevotellaceae_UCG_001* were the important taxa contributing to the differences in the rumen microbiota between the MN and CON groups (Table S7). A total of 60 biomarkers were found in the AGF and MN groups, and *Prevotella_1* and *Veillonellaceae* were the important taxa contributing to the differences in the rumen microbiota between the AGF and MN groups (Table S8).

#### 3.2.5. Microbiota Functional Profile Prediction

**Figure 6:**
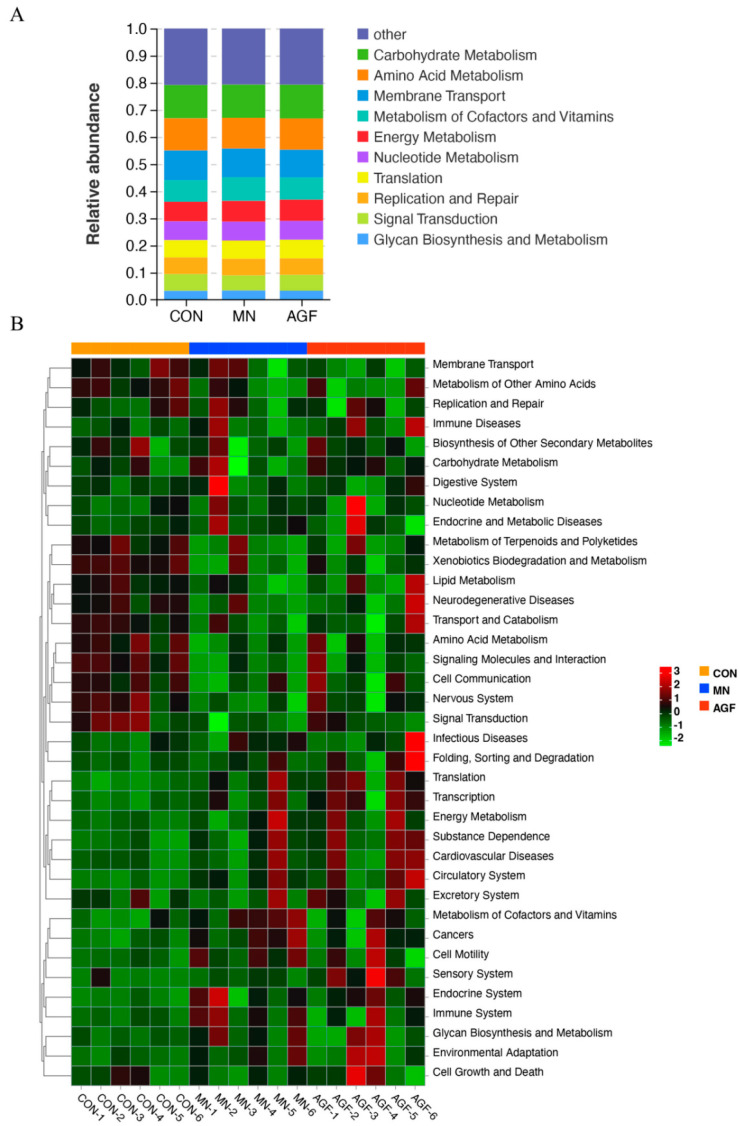
Prediction of microbiota functions by Tax4Fun Platform. (**A**) Level_2 stacked chart. (**B**) Level_2 heat map.

Functional prediction of the rumen microbiota was performed on the Tax4Fun platform. The top ten pathways enriched by rumen microbiome in three groups are shown in Figure 6A. At level_2, a total of 37 functional categories in the rumen microbiota of kids were predicted in this study (Figure 6B). There were seven functional categories in the AGF and CON groups with significant differences (*p* < 0.05). There were 12 functional categories in the MN and CON groups with significant differences (*p* < 0.05) (Table S9). In the AGF group, the pathways of metabolism of cofactors and vitamins, glycan biosynthesis, and metabolism and cell motility were more enriched than other pathways (Figure 6B). In the MN group, the pathways of carbohydrate metabolism, cell growth and death, folding, sorting and degradation, energy metabolism, translation, nucleotide metabolism, and infectious diseases were more enriched than the other pathways (Figure 6B).

### 3.3. Plasma Physiology and Biochemistry

At the end of the experiment, compared with the CON group, the concentration of HDL-CH in the AGF group was significantly increased, while the concentration of LDL-CH was significantly lower (*p* < 0.05). The concentration of HDL-CH and LDL-CH in the MN group was not significantly different compared to the AGF and CON groups (*p* > 0.05). The GLU concentrations of the AGF group were significantly increased compared to those of the CON group (*p* < 0.05). The UREA concentrations of the CON group were obviously lower than those of the AGF group (*p* < 0.05) and the MN group (*p* < 0.01). The ALP and GPT concentrations of the CON group were higher than those of the AGF and MN groups (*p* < 0.05). The GOT concentrations of the CON group were obviously higher than those of the AGF group (*p* < 0.05) and the MN group (*p* < 0.01) (Table 3).

**Figure 7:**
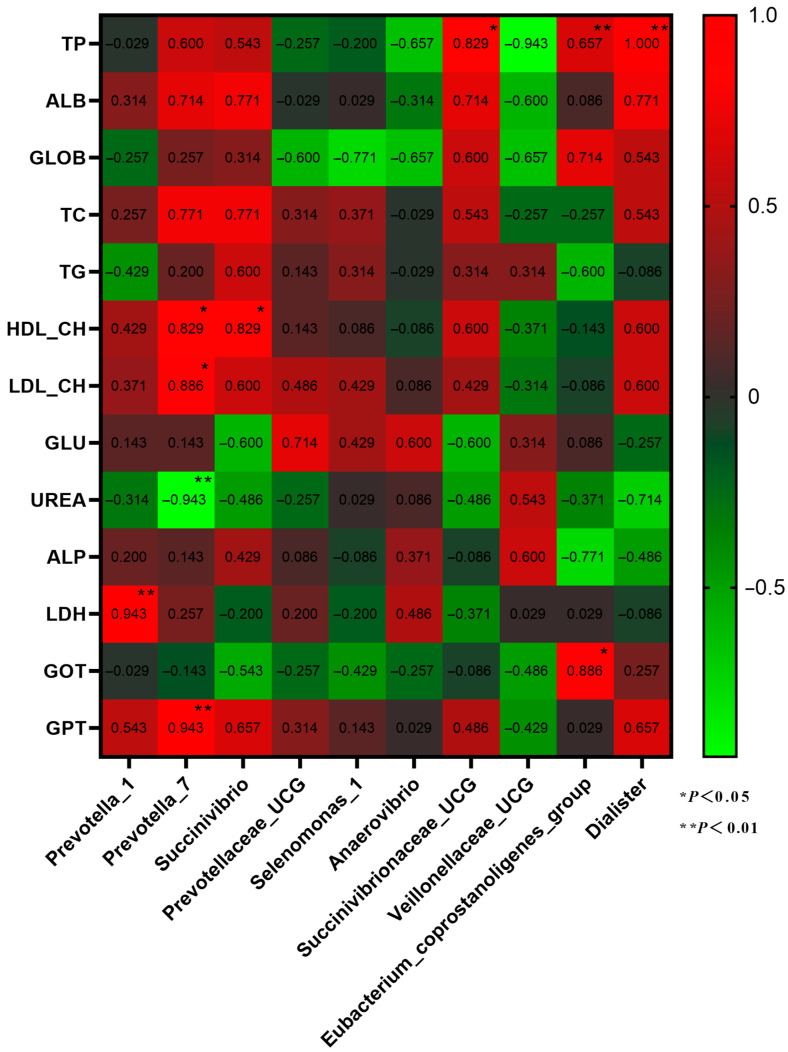
Correlation analysis between dominant genera of rumen microbiota and plasma physiology and biochemistry.

Correlation analysis was conducted on the dominant genera of rumen microbiota and plasma physiological and biochemical characteristics (Figure 7). *Prevotella_7* is significantly positively correlated with HDL-CH, LDL-CH, and GPT, and *Prevotella_1* is significantly negatively correlated with LDH (*p* < 0.05). *Eubacterium_coprostanoligenes_group* has a significant positive correlation with GOT and TP and a significant positive correlation between *Dialister* and TP (*p* < 0.01).

## 4. Discussion

With a pressing need for non-antibiotic interventions to enhance livestock health and productivity, attention has shifted towards natural phytochemicals such as flavonoids [39,40,41]. Among these, dihydromyricetin (DMY), the most abundant flavonoid compound derived from Ampelopsis grossedentata, has demonstrated a range of pharmacological effects encompassing cardioprotection, antidiabetic, hepatoprotection, neuroprotection, anticancer, and skin-protective properties [42,43]. In the absence of antibiotics, the emergence of alternative feed additives such as AGF holds promise. However, information on the use of AGF in ruminants remains scarce. This study, therefore, aims to assess the efficacy of AGF by evaluating its impact on the growth performance, rumen microbiota, and blood metabolites of kids, which serve as indicators of their growth and health status.

The rumen acts as a bioreactor for anaerobic microorganisms, with pH crucial for balancing fermentation and microbial metabolism. Optimal pH (6.2–6.8) facilitates fiber breakdown and volatile fatty acid production [44]. This study observed a pH trend (MN group > CON group > AGF group) without significant differences, and all were suitable for microbial survival. Adding AGF increased richness, evenness, and species diversity in kid rumen fluid. Beta-diversity analysis indicated AGF and monensin clustering, initially suggesting AGF as a potential monensin substitute.

This study found that *Bacteroidetes*, *Firmicutes*, and *Proteobacteria* constituted 95% of the rumen bacteria population, aligning with prior research [45,46]. These bacteria influence host metabolism and immunity [47]. Monensin reduced the anundance of *Proteobacteria*, diverging from some findings, possibly due to rumen bacterial community dynamics [48,49]. Concerning the addition of AGF to the diet, it exerted no significant impact on *Firmicutes*, yet it elevated the abundance of *Bacteroidetes* and curtailed *Proteobacteria*.

Further analysis found that AGF decreases the relative abundance of *Vibrionaceae* and *Succinivibrionaceae*. Several *Vibrionaceae* are infamous for their pathogenicity or symbiotic relationships [50]. *Succinivibrionaceae* are Gram-negative, spore-forming bacteria found in the gut microbiota that aid in food digestion and maintain gut health. Recent studies suggest that they may also play important roles in human gut health and metabolism, with some links to metabolic disorders and potential anti-inflammatory effects [51,52]. AGF notably elevates the relative abundance of *Ruminococcaceae*, an anaerobic Gram-positive bacterium within the *Firmicutes* phylum. *Ruminococcaceae* holds a pivotal role in the rumen microbial consortium of ruminants, contributing to the degradation of cellulose and lignin while generating short-chain fatty acids as an energy resource for the host animals. Intriguingly, the present investigation reveals that AGF and monensin concomitantly diminish the relative abundance of *Vibrionaceae*. An in-depth examination of *Vibrionaceae* uncovers a negative association with the relative abundance of *Lachnospiraceae*, a predominant gut bacterium implicated in mucin degradation [53], exhibiting a significant inverse correlation with obesity [54,55] and playing a critical function in preserving intestinal mucosal health. This study confirmed that the impact of rumen microbiota on the host is not singular, but instead intricate and multifaceted.

Within the complex microbial milieu, no single genus prevails. Nonetheless, additives significantly influence *Prevotella*, particularly strains *Prevotella_1* and *Prevotella_7*. *Prevotella* demonstrates fiber-degradation capacities in ruminant feces [56]. AGF reduces Succinivibrio abundance, a non-spore-forming, sugar-fermenting taxon generating metabolic byproducts, encompassing acids and CO_2_ [57]. This flavonoid selectively diminishes anaerobic bacteria, displaying inverse associations with anaerobic taxa, concurrently decreasing *Anaerovibrio* prevalence while enhancing it with monensin. Cumulatively, flavonoids exert a positive influence on bacterial community structure.

Differential microbiota among the three groups in this experiment were obtained by LEfSe analysis. *Prevotella_1* and *Prevotella_7* are the two differential bacterial genera that we are most concerned about, and AGF enables the relative abundance of *Prevotella_1*. There was a significant increase, while the relative abundance of *Prevotella_7* significantly decreased. A recent study also found that reduced levels of Prevotella may be involved in butyrate production [58], a key nutrient for the intestinal epithelial cells [59]. It has been demonstrated that *Prevotellaceae_UCG-001* is a beneficial bacterium [60]. Xue et al. found that the relative abundances of *Prevotella 1*, and *Prevotellaceae UCG-001* were significantly related to the majority of the dominant functions, which was similar to our study [61]. Thus, through promoting host health or energy metabolism, *Prevotella_1* was reported to increase the feed efficacy in pigs [62]. The relative abundance of *Prevotella_7* in membranous nephropathy patients is significantly higher than that of healthy people, so it can be used as a diagnostic biomarker [63]. It is suggested that the additive, Ampelopsis grossedentata flavonoids, can facilitate the proliferation of beneficial microbes.

The results of functional prediction showed that the number of microorganisms enriched in the metabolism of cofactors and vitamin signaling pathways in the diet supplemented with AGF was significantly higher than that in the diet without additives. Cofactors, the non-protein entities, facilitate enzyme-catalyzed reactions by modulating enzyme conformation or offering essential chemical groups through their binding to enzymes. Inorganic ions, coenzymes, and hormones are among the constituents of cofactors [64]. Vitamins, a subset of these, act as precursors to numerous coenzymes that participate in diverse biochemical reactions, including but not limited to energy metabolism, DNA synthesis, and immune system function [65,66]. Microbial species interact in vivo to form more complicated food chains, and some of these relationships center on glycan metabolism [67]. The studies have shown the relative abundance of the enrichment of the glycan biosynthesis and metabolism pathway in the diet supplemented with AGF. We speculate that the possible reason for the significant enrichment of the glycan biosynthesis and metabolism pathway is the abundance of certain bacteria, such as Fibrobacter and Ruminococcus, that fermented crude fiber increases.

Plasma metabolites not only constitute a robust dataset for elucidating the health status of animal organisms but also serve as vital indices for assessing feed utilization, nutrient absorption and metabolism, and alterations in organ function in animals [68]. In this study, the addition of Ampelopsis grossedentate flavonoids to the diet resulted in a significant augmentation of GLU content, furnishing kids with increased energy resources. Research has demonstrated that the inclusion of monensin in sheep feed can lead to substantial elevations in GLU, ALB, and GLOB concentrations [69]. In concordance with previous findings, this study also demonstrated consistent outcomes upon the inclusion of monensin. UREA serves as a proxy for the metabolic state of nitrogenous compounds within the animal organism and functions as an indicator of feed protein utilization efficiency. Elevated concentrations correspond to diminished protein utilization efficiency. Further evaluation is necessary to determine the specific reasons for the observed increase in plasma UREA concentration resulting from the additives utilized in this study. Typically, reduced levels of GOT, GPT, and ALP in the plasma are considered favorable, as heightened levels may signify liver or other tissue damage or disease. The findings of this study demonstrate that AGF has significant potential as a novel feed additive, as evidenced by its ability to effectively reduce the levels of these indicators. In this study, compared with the CON group, the plasma LDL-CH content in the AGF group was significantly reduced, while the LDL-CH content showed the opposite trend. HDL-CH transports lipids from tissues to the liver and is thus known as “good cholesterol,” whereas LDL-CH is largely related to the low plasma cholesteryl esters transfer activity and thus known as “bad cholesterol.” Changes of LDL and HDL for ruminant animals have an impact on lipid absorption and transport [70,71]. In brief, Ampelopsis grossedentate flavonoids improve liver function and lipid absorption and transport, thereby enhancing the immune and metabolic function of kids.

A study has demonstrated that the incorporation of flavonoids into the dietary regimen of rabbits has led to improved muscle oxidation stability, without any deleterious impact on the animals’ growth performance [72]. Furthermore, subsequent investigations have revealed that the supplementation of alfalfa flavonoids in the diet of broiler chickens led to a marked decline in feed-to-gain ratio by 2.98% to 16.53% compared to the control group, while also enhancing the survival rate of the broilers [73]. The present study’s empirical results align with prior research, whereby the supplementation of AGF has exhibited a noteworthy improvement in the final weight, daily feed intake, and average daily gain of kids. In addition, this intervention has been found to substantially diminish the feed-to-meat ratio by 38.7%. It is postulated that such favorable outcomes may be attributed to the flavonoids’ potential to mitigate the oxidative stress response in kids, ameliorate intestinal injury, and foster the growth and development of the intestines [8]. The improvement effect of monensin on the growth performance of ruminants has been studied for a long time [74,75,76,77], and the similar effect of AGF and monensin in improving kid growth performance has been observed to be comparable. In the current study, a notable upsurge in daily weight gain was documented for the MN group in comparison to the CON group. These empirical findings lend indirect support to the beneficial impact of AGF on kid growth performance, implying the feasibility of substituting antibiotic additives, such as monensin. Hence, this study suggests that the integration of AGF in animal feed can elicit favorable outcomes in kids, characterized by marked enhancements in weight gain and feed utilization efficiency and a reduction in feed-to-gain ratios.

## 5. Conclusions

**Figure 8:**
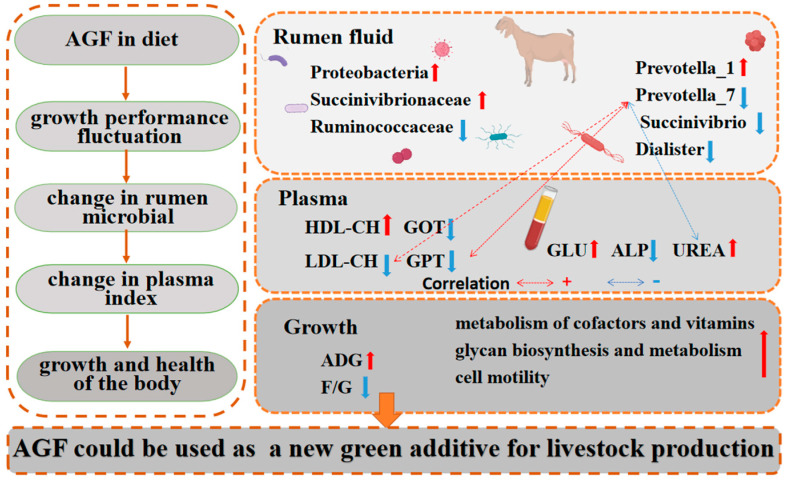
Ampelopsis grossedentata flavonoids regulate the composition of rumen microbial in kids, thereby improving growth performance and body health. The red arrows represent upward adjustments and blue arrows represent downward adjustments. The red dashed lines represent positive correlations, and the blue dashed lines represent negative correltions.

A regulation diagram drawn based on the results is shown (Figure 8). In conclusion, Ampelopsis grossedentata flavonoids in the diet improve kids’ rumen microbial diversity and richness, optimize microbial communities, and improve rumen health. Ampelopsis grossedentata flavonoids in the diet also improve kids’ plasma physiology and biochemistry, as well as the liver function and immunity. Of note, the relative abundance of *Prevotella_7* is decreased, which was found to exhibit a positive correlation with GPT and LDL-CH. These effects significantly reduce the feed-to-meat ratio of kids. To a certain extent, AGFs have the potential to replace monensin and serve as a promising environmentally friendly feed additive. Thus, Ampelopsis grossedentata flavonoids could be used as an important new green additive in the livestock production industry.

## Figures and Tables

**Table 1:** Composition and nutrient level of the basal diet (dry matter basis).

Material	Content (%)	Nutrients	Content
Corn	30.00	Digestible energy (MJ/Kg)	12.57
DDGS	8.00	Crude protein	14.07
Fatty powder	5.00	EE	3.11
Grass meal	55.00	CF	16.83
CaHPO_4_	0.50	NDF	27.42
NaCl	0.50	ADF	21.17
Premix	1.00	Calcium	1.14
Total	100.00	Total phosphorus	0.34

Note: The premix provided the following per kg of diets VA: 15,000 IU, VD: 2200 IU, VE: 50 IU, Fe: 55 mg, Cu: 12.5 mg, Mn: 47 mg, Zn: 24 mg, Se: 0.5 mg, I: 0.5 mg, Co: 0.1 mg.

**Table 2:** Effects of different additives on the growth performance of kids.

Items	CON Group	MN Group	AGF Group
IBW (kg)	14.18 ± 1.84	14.88 ± 1.19	14.69 ± 2.06
FBW (kg)	19.80 ± 1.66 ^b^	22.13 ± 1.97 ^a^	22.51 ± 3.50 ^a^
ADFI (g)	683.44 ± 21.29	686.85 ± 21.66	699.15 ± 21.83
ADG (g)	62.43 ± 10.39 ^a^	80.63 ± 16.20 ^b^	86.77 ± 20.29 ^b^
F/G	13.77 ± 4.91 ^a^	9.05 ± 1.64 ^b^	8.44 ± 1.13 ^b^

Note: The same or no letters on the shoulder indicate no significant difference (*p* > 0.05), different lowercase letters indicate significant difference (*p* < 0.05).

**Table 3:** Effects of different additives on plasma physiology and biochemistry of kids.

Items	CON Group	MN Group	AGF Group
TP (g/L)	62.28 ± 1.54	65.33 ± 1.95	60.21 ± 3.34
ALB (g/L)	26.05 ± 0.71	26.66 ± 0.47	24.40 ± 1.61
GLOB (g/L)	36.23 ± 1.51	38.66 ± 2.14	35.81 ± 2.47
TC (mmol/L)	1.79 ± 0.16	1.72 ± 0.14	1.76 ± 0.20
TG (mmol/L)	0.30 ± 0.05	0.25 ± 0.05	0.27 ± 0.08
HDL-CH (mmol/L)	0.93 ± 0.07 ^a^	1.0 ±0.08 ^ab^	1.26 ± 0.14 ^b^
LDL-CH (mmol/L)	0.95 ± 0.12 ^a^	0.73 ± 0.08 ^ab^	0.64 ± 0.09 ^b^
GLU (mmol/L)	3.02 ± 0.19 ^a^	3.32 ± 0.15 ^ab^	3.62 ± 0.10 ^b^
UREA (mmol/L)	2.95 ± 0.47 ^Aa^	4.66 ± 0.35 ^Bb^	4.41 ± 0.30 ^b^
ALP (U/L)	449.00 ± 106.84 ^a^	206.50 ± 31.29 ^b^	192.43 ± 60.37 ^b^
LDH (U/L)	238.83 ± 7.89	257.88 ± 21.67	270.71 ± 17.83
GOT (U/L)	110.50 ± 11.86 ^A^	68.63 ± 4.42 ^B^	85.14 ± 12.94 ^AB^
GPT (U/L)	17.50 ± 1.28 ^a^	13.25 ± 1.44 ^b^	13.00 ± 1.23 ^b^
GOT/GPT	6.40 ± 0.66	5.52 ± 0.50	7.58 ± 2.30

Note: The same or no letters on the shoulder indicate no significant difference (*p* > 0.05), different lowercase letters indicate significant difference (*p* < 0.05), and different capital letters indicate extremely significant difference (*p* < 0.01).

## Data Availability

All raw sequences were deposited in the NCBI Sequence Read Archive (https://www.ncbi.nlm.nih.gov/, accessed on 2 March 2023) under accession number SRA Accession no. PRJNA960633.

## Supplementary Materials

The following supporting information can be downloaded at: https://www.mdpi.com/article/10.3390/ani13152454/s1, Table S1: Data preprocessing statistics and quality control. Table S2: Good’s coverage values of all samples. Table S3: Relative abundance (%) of dominant microbiota phylum in the rumen of kids. Table S4: Relative abundance (%) of dominant microbiota family in the rumen of kids. Table S5: Relative abundance (%) of dominant microbiota genera in the rumen of kids. Table S6: LEfSe analysis in the AGF and CON groups. Table S7: LefSe analysis in the MN and CON groups. Table S8: LefSe analysis in the AGF and MN groups. Table S9: Abundance of functional predictive heat map.
